# Features of PABIG^nx^ 3D Polymer Gel as an Ionising Radiation Dosimeter

**DOI:** 10.3390/ma15072550

**Published:** 2022-03-31

**Authors:** Marek Kozicki, Malwina Jaszczak, Piotr Maras

**Affiliations:** 1 Department of Mechanical Engineering, Informatics and Chemistry of Polymer Materials, Faculty of Materials Technologies and Textile Design, Lodz University of Technology, 90-543 Lodz, Poland; malwina.jaszczak@p.lodz.pl; 2 Department of Radiotherapy Planning, Copernicus Hospital, 90-543 Lodz, Poland; p.maras@kopernik.lodz.pl

**Keywords:** PABIG^nx^, 3D polymer gel dosimetry, radiotherapy dosimetry, ionising radiation

## Abstract

This work presents the features of the PABIG^nx^ 3D polymer gel dosimeter. It consists of two cross-linkers: poly(ethylene glycol) diacrylate (PEGDA), as one biacrylic component, and *N*,*N*′-methylenebisacrylamide (MBA), which is another cross-linker often used in 3D dosimeters. Additionally, it contains oxygen scavenges of copper sulfate pentahydrate and ascorbic acid. All ingredients are embedded in a physical gel matrix of gelatine. Upon irradiation, the biacrylic cross-linking agents (PEGDA and MBA) undergo radical polymerisation and cross-linking, which is manifested by the appearance of the opacity of the intensity related to the absorbed dose. PABIG^nx^ was irradiated with an oncological source of ionising radiation, and analysed by using a nuclear magnetic resonance (0.5 T). The following characteristics were obtained: (i) linear and dynamic dose-response of 0.5 to ~18 Gy and 40 Gy, respectively, (ii) dose sensitivity of 0.071 ± 0.001 Gy^−1^ s^−1^, (iii) integral 3D dose distribution for at least 24 days after irradiation, (iv) adequate batch-to-batch reproducibility, (v) dose-response independent of irradiation with 6 MV photons, 15 MV photons, 6 MV photons FFF of 0.0168–0.1094 Gy/s dose rates, and (vi) soft tissue equivalence. The study showed that the features of PABIG^nx^ confirm its suitability for use in 3D radiotherapy dosimetry.

## 1. Introduction

Since the 1980s, a number of 3D dosimeters for radiotherapy applications have been proposed and thoroughly tested. These include both the first Fricke 3D dosimeters with magnetic resonance imaging (MRI) reading and their successors such as polymer gel dosimeters, radiochromic gels and plastics. The most important aspects of 3D polymer dosimetry are discussed elsewhere [1] (and references therein). Other 3D dosimeters such as radiofluorogenic gels [2], polyurethane resin-based radiochromics [3,4,5], silicone-based dosimeters [6], radiochromic gels based on micellar solutions [7,8,9,10,11], gels containing tetrazolium salt or leuco dyes, and Pluronic F-127 physical gel matrix [12,13,14,15] were also investigated.

In this study, PABIG^nx^ 3D polymer gel dosimeter is examined. The acronym of this dosimeter comes from letters selected from the names of the dosimeter ingredients, as follows: Poly(ethylene glycol) diAcrylate, *N*,*N*′-methylenebisacrylamide, which is abbreviated to Bis, hence BIs, Gelatine, and “nx” superscript denotes “normoxic”, which means “prepared in the presence of oxygen” and contains oxygen scavengers. This dosimeter is the successor of the PABIG polymer gel dosimeter, which was first announced in 2003 [16] and investigated in brachytherapy [17] and radiosurgical beam dosimetry [18]. Dosimeters from the PABIG family contain two cross-linking agents, which is unique in 3D dosimetry: poly(ethylene glycol) diacrylate (PEGDA) and *N*,*N*′-methylenebisacrylamide (MBA). Other dosimeters contain one cross-linker and one acryl/vinyl monomer or an acrylic acid [1]. MBA is often used in other 3D polymer gel dosimeters [1]. The application of PEGDA for the manufacturing of gel dosimeters was the result of the search for a substitute for the toxic acrylamide used in the preparation of the first polymer gel dosimeters [1]. The PABIG dosimeter required, however, tedious saturation with an inert gas (argon) in order to eliminate the influence of oxygen on the radical polymerisation and cross-linking under the influence of ionising radiation; these are the reactions used to record the 3D dose distribution in polymer gel dosimeters. On the other hand, PABIG^nx^ contains oxygen scavengers and does not require saturation with inert gas, which facilitate its preparation. It was first announced elsewhere [19] following a proposal from Fong et al. These authors showed the preparation of 3D polymer gel dosimeters with oxygen scavengers [20]. The composition of PABIG^nx^ and its response to dose were reported after irradiation with a technical source of ionising radiation (^60^Co) and by reading with the nuclear magnetic resonance (NMR, 0.5 T) [19]. It was shown that this dosimeter was stable from 20 to at least 95 h after irradiation; typically, polymer gel dosimeters stabilize up to about 24 h after irradiation, and PABIG, the predecessor of PABIG^nx^, also stabilizes after about 20 h after irradiation. PABIG^nx^ saturated after absorption of about 40 Gy, its dose threshold was lower than 1 Gy, the linear dose range was about 18 Gy, and the dose sensitivity was ~0.1 Gy^−1^ s^−1^ [19]. Additionally, PABIG^nx^ was calibrated using a medical linear accelerator, a ^192^Ir HDR brachytherapy source and a 1.5 T MRI readout [21]. This publication presents a comprehensive approach to calibrating PABIG^nx^ using cross beam, multi vial, depth dose and brachytherapy calibration methods. These methods use containers adapted to polymer gel dosimeters and individual calibration methods, specific conditions and protocols for irradiation and MRI scanning, and the MRI results are appropriately processed using a dedicated software package, for instance, polyGeVero^®^ and polyGeVero^®^-CT (GeVero Co., Lodz, Poland). Overall, the PABIG^nx^ dose-response was similar for the multi vial, cross beam, and depth dose calibration, with the dose sensitivity being 0.0898, 0.0971 and 0.0975 Gy^−1^ s^−1^, respectively. The dose sensitivity for brachytherapy calibration was slightly lower and amounted to 0.0787 Gy^−1^ s^−1^. It was found that the linear dose range does not exceed 18 Gy, and the dynamic dose response does not exceed 40 Gy, which is consistent with the previous study [19]. No further studies have been performed with the PABIG^nx^ gel dosimeter.

In order to use PABIG^nx^ in radiotherapy dosimetry, including the verification of treatment planning generated plans of irradiation of tumors or similar applications, this dosimeter needed further analysis. Therefore, the novelty of the present study was to test the PABIG^nx^ dosimeter with a 0.5 T NMR reading to find the following: (i) batch-to-batch reproducibility, (ii) long-term stability, (iii) tissue equivalence, and (iv) the effect of the dose rate and radiation energy on the dosimeter dose performance.

## 2. Materials and Methods

### 2.1. Preparation of PABIG^nx^

The PABIG^nx^ polymer gel dosimeter was prepared analogously to the previous preparation scheme [21]. In brief, poly(ethylene glycol) diacrylate (PEGDA, M_n_ = 700 g mol^−1^; Sigma-Aldrich, Saint Louis, MO, USA), *N*,*N*′-methylenebisacrylamide (MBA or Bis; Sigma-Aldrich, Saint Louis, MO, USA), gelatine (type A, 300 Bloom; Sigma-Aldrich, Saint Louis, MO, USA), copper sulfate pentahydrate (CuSO_4_ · 5H_2_O; Chempur, Piekary Śląskie, Poland), ascorbic acid (AsAc; Chempur, Piekary Śląskie, Poland) and distilled water were used. First, MBA (4% *w*/*v*) was dissolved at 45 °C, then, gelatine (5% *w*/*v*) was added in portions and the mixture was stirred until it turned clear and transparent. After that, it was cooled to about 30 °C for the addition of PEGDA (4% *w*/*v*). Finally, copper sulfate pentahydrate (0.0004% *w*/*v*) and ascorbic acid (0.007% *w*/*v*) were added. After thorough mixing, the liquid composition was poured into nuclear magnetic resonance (NMR) glass vials (0.63 mm wall thickness, 8.75 mm inner diameter, ~100 mm length) and sealed with rubber caps and Parafilm^®^. The dosimeter samples, after preparation, were kept at room temperature and protected from daylight.

### 2.2. Irradiation of PABIG^nx^

The irradiation of PABIG^nx^ took place about 48 h after preparation. This was sufficient time to let the dosimetric solution convert into a physical gel (usually about 24 h is enough for this composition to convert into a solid physical gel). The physical gel is understood to be hydrophilic polymer macromolecules that are joined by other bonds and interactions than covalent bonds, with water molecules embedded in their structure. Such gels are reversible, which means that upon external stimuli (for instance, temperature, pH or ionic strength change) they can convert to solutions. A simple flow test can be performed to determine if the dosimetry solution has converted to a physical gel. For this purpose, the sample with the dosimeter should be stored at room temperature and tilted at an angle of 45°. If the dosimeter is still liquid, it will flow; if not, it will remain rigid and its surface will be parallel to the bottom of the container. In this way, the time (or storage temperature) required to convert the dosimetric solution into a dosimetric gel can also be determined. A more sophisticated method would be, for instance, differential scanning calorimetry (DSC), which allows for the precise determination of the phase transition for physical gels or 3D gel dosimeters (see Figure 3 in [22]). Note that heating to more than 30 °C will convert the physical gel dosimeter to a liquid (sol) dosimeter.

The dosimeter was irradiated with a medical accelerator emitting X-ray radiation (TrueBeam, Varian, Palo Alto, CA, USA). The samples were placed in a water phantom (GeVero Co., Lodz, Poland) 5 cm below the water level so that the SSD was equal to 95 cm, the irradiation field was set to 2 × 2 cm^2^ (defined by jaws of the collimator; collimator = 0°, gantry = 0°), and the following dose rates were used for specific radiation beams: X6, 100 MU/min (0.0168 Gy/s); X6, 300 MU/min (0.0505 Gy/s); X6, 600 MU/min (0.101 Gy/s), X15, 600 MU/min (0.1094 Gy/s), and X6 FFF, 600 MU/min (0.0974 Gy/s) (FFF denotes Flattening Filter Free). The dosimeter samples after irradiation were kept at room temperature and protected from daylight.

### 2.3. NMR Measurements

T_2_ nuclear magnetic resonance (NMR) measurements were employed to track the changes in the irradiated PABIG^nx^ polymer gel dosimeters. A benchtop NMR relaxometer (Minispec 20 MHz, Bruker, Billerica, MA, USA) was used. The first measurements were carried out 24 h after irradiation to allow for termination of the post-irradiation effect; the measurements were performed at 25 °C (guaranteed by a heating–cooling system, Haake, Vreden, Germany; heating-cooling proton-free liquid: Fluorinert^TM^ FC-43, 3M Company, Kajetany, Poland). The NMR measurements were continued up to 11 days after irradiation. The sequence was as follows: the time delay between consecutive pulses was 80 ms; the first time-to-echo (TE) was equal to 80 ms; 65 data points were received (TE range: 80–5200 ms; each datapoint was a mean value of 16 measurements). The data was further processed employing only 63 data points after discarding the first two as the two clearly deviated from the other points [21,23]. The points were fitted on the basis of the first-order exponential of free induction decay (FID) to calculate the T_2_ (spin-spin relaxation time). Next, T_2_ values were converted to R_2_ (relaxation rate; R_2_ = 1/T_2_) and discussed as a function of radiation dose.

### 2.4. Tissue/Water Equivalence

The density (ρ) of the PABIG^nx^ polymer gel dosimeter and the elemental composition (% by weight) were obtained in the former study and were equal to 1.02 g cm^−3^ at 23 °C and 10.838 ^1^H, 7.399 ^6^C, 1.777 ^7^N, 79.986 ^8^O, 9.98 × 10^−7 29^Cu, and 5.03 × 10^−7 16^S, respectively [21]. The density was also measured in this study, however, at 21 °C, which gave a similar value of 1.017 ± 0.002 g cm^−3^ (based on weighting of eight 10 mL flasks filled in with the dosimeter). The following calculations were also performed: effective atomic number (Z_eff_), atomic-to-mass number ratio (<Z/A>) and electron density (ρ <Z/A>), based on the approach reported elsewhere [24]. Mass attenuation coefficients as a function of radiation energy for PABIG^nx^ and water were calculated using the XCOM Program of the National Institute of Standard and Technology (NIST, Gaithersburg, MA, USA).

### 2.5. Reproducibility

Three batches of PABIG^nx^ were prepared as described in Section 2.1 to test the reproducibility of the manufacturing, the procedure of irradiation and the NMR measurements. After both preparation, irradiation and NMR measurements, the samples were stored under protection from daylight at room temperature (about 21 °C). They were irradiated 48 h after preparation and measured 24 h after irradiation. The irradiation conditions were the same as described in Section 2.2; however, the beam characteristics were as follows: X6, 600 MU/min (0.101 Gy/s). The NMR measurements were performed as described in Section 2.3.

### 2.6. Temporal Stability Study

The PABIG^nx^ batch was prepared as described in Section 2.1. It was irradiated under the conditions described in Section 2.2; the beam characteristics were as follows: X6, 600 MU/min (0.101 Gy/s). Following that, they were measured as described in Section 2.3, 24 h to 11 days after irradiation. After preparation and between measurements, PABIG^nx^ samples were stored in the dark at room temperature (about 21 °C).

### 2.7. Spatial Stability

The PABIG^nx^ batch was prepared as described in Section 2.1. It was irradiated under the conditions described in Section 2.2; the beam characteristics were as follows: X6, 600 MU/min (0.0794 Gy/s). Glass vials were filled in with the dosimeter (dimensions: 30 mm in diameter and 100 mm height), which were tightly closed with a plastic cap and Parafilm^®^. One vial served as background and three were irradiated roughly in the center so as to obtain a 1 cm thick slice of the irradiated zone below and above which the non-irradiated part of the dosimeter remained. Two samples were irradiated to the dose of 10 and 15 Gy, which is within the linear dose range of PABIG^nx^, whereas one sample was irradiated to the dose of 40 Gy, which is above the saturation point of this dosimeter.

After irradiation, the samples were transferred from the radiotherapy department to another laboratory for optical analysis. They were placed and stored in a cabinet (Spencer, Holland) equipped with a D65 illuminant. D65 illuminant is considered standard illuminant as defined by the International Commission on Illumination (CIE). It stands for standard outdoor illumination conditions in different parts of the world. The spectrum of the illuminant was measured and discussed elsewhere [25]. To eliminate imperfections in the cabinet walls and provide adequate background for the irradiated samples, its inferior was covered with black paper sheets. The photographs of the irradiated PABIG^nx^ were taken periodically with a Nikon reflex digital camera (Nikon D5100, NIKKOR lens DX, AF-S 18–105 mm, Tokyo, Japan), which was set at a fixed position in front of the samples, and the D65 illuminant was switched on for a few seconds to take a photo. The photographs were taken for the 24 days after irradiation. Then, they were processed with ImageJ (National Institutes of Health, Bethesda, MD, USA).

## 3. Results and Discussion

### 3.1. Batch-to-Batch Reproducibility

Three separate series of PABIG^nx^ polymer gel dosimeter were prepared, irradiated and measured with the aid of NMR. This was done by keeping the preparation procedure, irradiation conditions and NMR measurement as close as possible to each other each time. It is well-known that the final calibration data for a 3D dosimeter depends on a number of conditions, including: (i) the precision of dosimeters preparation; even small differences in component concentrations or proportions (ratio) between co-monomers can affect major features such as dose sensitivity, while incorrect concentrations of oxygen scavengers will increase dosimeter dose threshold, (ii) variable temperature history of a 3D dosimeter during preparation and storage, which may induce some undesirable changes to the dosimeter; for instance, storing a polymer gel dosimeter at low temperature may initiate crystallization of some monomers while high temperature may convert a physical gel matrix into liquid, (iii) exposure to daylight, which may induce polymerisation of co-monomers and thus reduce the range of the dosimeter dynamic dose response, (iv) imprecise filling the vials with a dosimeter, leaving empty air voids and inaccurate sealing of the vials to protect against oxygen infusion may cause differences in dosimeter response to irradiation, because the constant oxygen infusion will interfere with the radical polymerisation and cross-linking, (v) large fluctuations of the dosimeter temperature during irradiation, which may adversely affect both the spatial stability of recorded dose distribution and the effect of polymerisation and cross-linking, and (vi) temperature changes for batches measured with NMR (or MRI), which will affect the T_2_ spin-spin relaxation time, and, consequently will result in a different R_2_ vs. dose calibration relation.

The assumed hypothesis of the experiment was that a conclusion about good reproducibility would be reached if the relationship of the relaxation rate with the radiation dose for the three sets of experiments (preparation, irradiation, measurement) were the same or very similar (approximately within the standard deviations for the points) without clear and striking deviations of the R_2_ values for the PABIG^nx^ batches.

**Figure 1:**
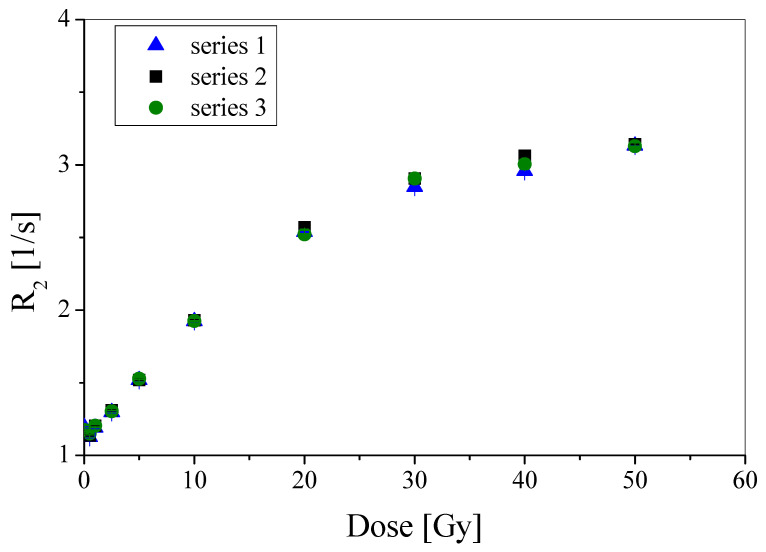
Repeatability of PABIG^nx^ dosimeter expressed as a relation between R_2_ and radiation dose for three series of this dosimeter measured 24 h after irradiation. The R_2_ values are given with the standard deviation calculated from T_2_ measurements.

The results presented in Figure 1 show good reproducibility of the manufacturing, irradiation and NMR measurement procedures. The calibration points in the graph overlap within the standard deviations. Therefore, the studies reported below were pursued with only one batch of PABIG^nx^. Moreover, the obtained calibrations (Figure 1) showed that the PABIG^nx^ dose threshold was 0.5 Gy or less (0.5 Gy was the lowest dose applied in this study), the linear dose range is below ~18 Gy, the dynamic dose-response is up to about 40 Gy, and the dose sensitivity is 0.071 ± 0.001 Gy^−1^ s^−1^ (slope of linear regression; mean value out of three measurements). These values are in line with the previous findings [19,21], but for the dose sensitivity, which is slightly lower. It should be noted that the calibration conditions for the previous studies and the current study are not the same where, most importantly, the dosimeter reading was taken with the same NMR and pule sequence [19], however, the irradiation in [19] was performed using a ^60^Co technical source, but in the current study it was a high precision medical linear accelerator and the vials were placed in a water phantom to provide adequate radiation scattering conditions. In this study, the basic calibration parameters of PABIG^nx^ are also discussed below after long-term NMR measurements.

### 3.2. Impact of Dose Rate

The radical polymerisation of monomers can be described by the following Equation (1) [26]:

(1) $$-\frac{d\left[M\right]}{dt}=k_{p}\times k_{t}^{-0.5}\times P^{0.5}\times G_{M}^{0.5}\times {\left[M\right]}^{1.5}\times {\left(1+\frac{G_{s}\times \left[S\right]}{G_{M}\times \left[M\right]}\right)}^{0.5}$$

where [*M*] and [*S*] are monomer and solvent concentrations, respectively; *k_p_* and *k_t_* are the rates of propagation and termination, respectively; *P* is the dose rate; and *G_M_* and *G_S_* are the radiation yields of radicals formation on monomer and solvent, respectively. According to this equation, the polymerisation rate is related to, inter alia, the dose rate and the monomer concentration. Therefore, some studies examine the impact of monomer concentrations on the performance of polymer gel dosimeters [27]. Also, each polymer gel dosimeter, which contains vinyl/acryl monomers undergoing radical polymerisation by ionising radiation, can perform differently when irradiated with different dose rates. Thus, it was reasonable to evaluate the performance of PABIG^nx^ when irradiated with different dose rates that are typically used in radiotherapy of tumours.

**Figure 2:**
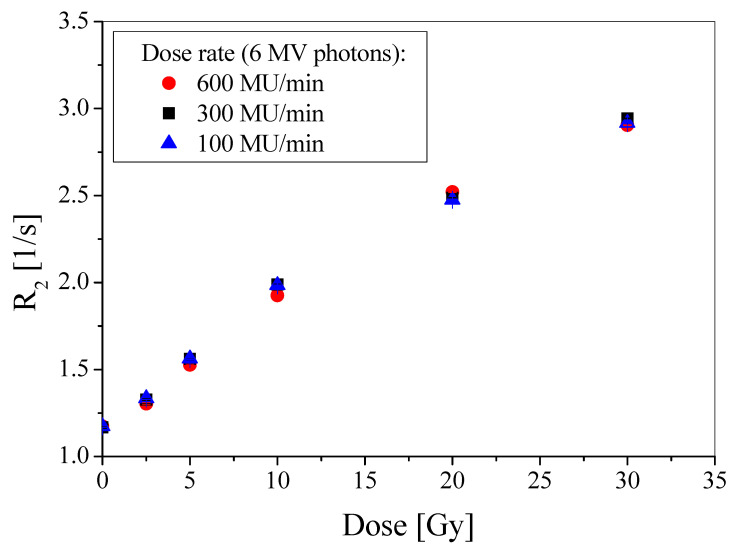
Dose-response repeatability of PABIG^nx^ dosimeter expressed as a relation between R_2_ and absorbed dose for three series of this dosimeter measured 24 h after irradiation. The R_2_ values are given with the standard deviations calculated from T_2_ measurements.

The results presented in Figure 2 show that the R_2_ = f(dose) relations for PABIG^nx^, which was irradiated with 6 MV photons of different dose rates (100 MU/min corresponding to 0.0168 Gy/s; 300 MU/min corresponding to 0.0505 Gy/s and 600 MU/min corresponding to 0.101 Gy/s), superimposed and the calibration points overlap within the standard deviations. This means that PABIG^nx^ is insensitive to changes in the selected narrow dose rate range. It should be noted, however, that this does not mean that the PABIG^nx^ dose-response would be the same at much higher or lower dose rates. This has not been investigated in this work.

### 3.3. Impact of Radiation Energy

**Figure 3:**
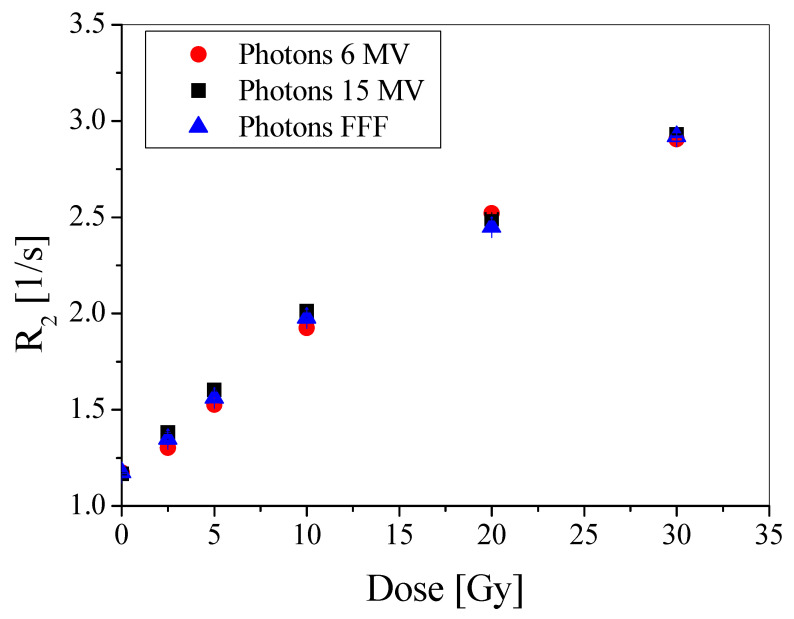
The relation of the relaxation rate (R_2_) versus absorbed dose for PABIG^nx^ dosimeter irradiated with 6 MV photons, 15 MV photons and 6 MV photons FFF. The samples were measured 24 h after irradiation. The R_2_ values are given with the standard deviation calculated from T_2_ measurements.

Different beams of radiation are used in the radiotherapy of tumors. Thus, it was necessary to examine the dose-response of the PABIG^nx^ polymer gel dosimeter for the irradiation with photons of 6 and 15 MV, and 6 MV FFF. The results are presented in Figure 3. The points on the graph overlapped within the standard deviations, which means that PABIG^nx^ was insensitive to changes between the chosen radiation beams. It should also be noted that the dose rates for 15 MV photons was equal to 0.1094 Gy/s and for 6 MV it was 0.0974 Gy/s, which in both cases is close to the dose rate of 6 MV photons (0.101 Gy/s) used in the reproducibility experiment. Thus, one can again conclude on the good reproducibility of manufacturing, irradiation and measurements of PABIG^nx^, apart from the independence of its dose-response from the dose rate and radiation beams in the chosen dose rate ranges and beam parameters.

### 3.4. Stability after Irradiation

**Figure 4:**
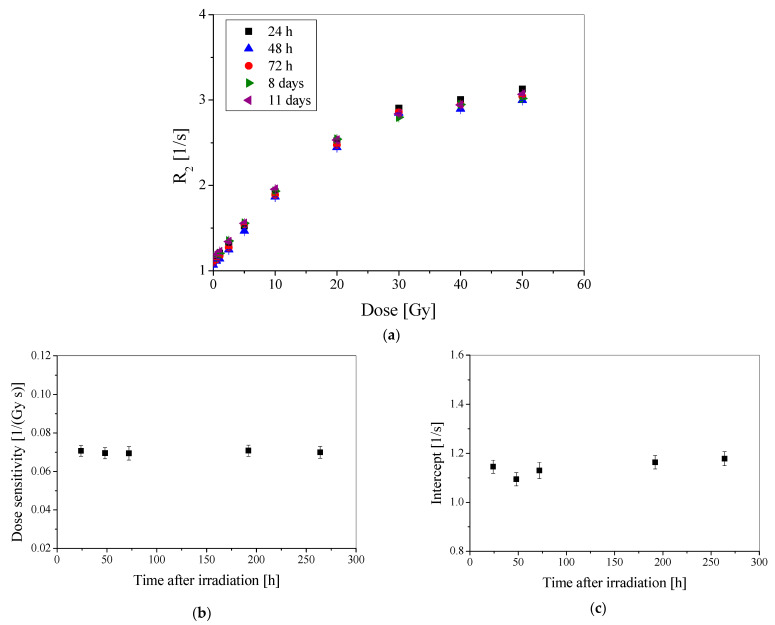
Stability over time of PABIG^nx^ dosimeter expressed as R_2_ dose-response measured from 24 h to 11 days after irradiation (**a**), calculated dose sensitivity (**b**), and calculated intercept (**c**).

After more than thirty years of research, it is basically known that 3D polymer gel dosimeters can be stable after preparation and irradiation for months or even years. Nevertheless, any dosimeter to be used in radiotherapy should be examined for long-term stability. Therefore, a batch of PABIG^nx^ was manufactured, irradiated and measured with the aid of NMR over-time after irradiation. The measurements were carried out until the eleventh day after irradiation. The following results were obtained: R_2_ = f(dose) (Figure 4a), dose sensitivity (Figure 4b) and intercept (Figure 4c).

In general, the dosimeter is stable over the chosen measurement period. This means that its measurements could be postponed for more than a week and the recorded dose would still remain the same; its stability is also assumed to last more than eleven days. Long-term stability may be important when measurements are carried out in a remote location from the irradiation, which would require several days of transfer of the dosimeter. This was the case for another study [28], in which different polymer gel dosimeters were used for verification of a treatment planning generated plan of irradiation of a tumor requiring irradiation in a radiotherapy department in one country and then transfer to another country for MRI measurements. In such cases, the long-term stability of the polymer gel dosimeters is essential.

The obtained results (Figure 4) were used to calculate the basic calibration characteristics (Table 1). The parameters in the table are the mean values of the five NMR measurements over-time after irradiation. The dose sensitivity is equal to 0.070 Gy^−1^ s^−1^, the dose threshold is 0.5 Gy, however, it was the lowest dose applied in this study, the linear dose range does not exceed ~18 Gy, and the dynamic dose range is up to about 40 Gy. Above this dose, PABIG^nx^ saturates and R_2_ does not increase. These findings are in line with what was found in Section 3.1.

### 3.5. Spatial Stability

**Figure 5:**
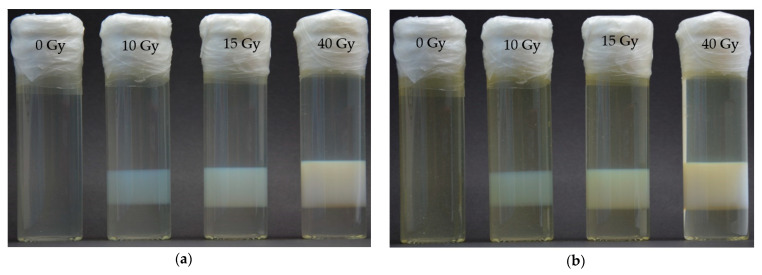
Photographs of PABIG^nx^ after inhomogeneous irradiation to dose of 0, 10, 15 and 40 Gy (**a**,**b**), where the photograph (**a**) was taken two hours after irradiation and the photograph (**b**) was taken on the 24th day (581 h) after irradiation.

The results of the PABIG^nx^ dose distribution spatial stability are shown in Figure 5. One vial was not irradiated and served as a blank sample to observe the chemical stability of the dosimeter. Any undesirable changes to the dosimeter in this vial would result in opacity due to the self-polymerisation and cross-linking of the dosimeter components, or yellowing due to degradation of gelatine during storage [22]. During the 24 days of storage of the blank sample, no opacity and no strong yellowing were observed, and the same observation was made for the non-irradiated parts of the irradiated dosimeter samples (Figure 5a,b). With the naked eye, a slight increase in the yellow color of the samples was observed, which indicates slow aging of the gelatin. This effect made it impossible to directly compare the profiles for the irradiated samples stored over time after irradiation. Observations of the irradiated parts of the samples yielded the following conclusions: (i) the thickness of the irradiated parts was higher at the higher doses (when measured two hours after irradiation, it was approximately 1.6, 1.8 and 2.1 mm for 10, 15 and 40 Gy, respectively). This was caused by a higher dose delivered to the penumbra region, which resulted in a stronger opacity of that region, which in turn widened the white irradiated region; (ii) the opacity (the intensity of the white colour of the irradiated regions) increased with increasing dose absorbed by the dosimeter. This is a well-known effect resulting from the higher degree of polymerisation and cross-linking of PABIG^nx^ monomeric components at higher doses [19], (iii) there was no diffusion of the irradiated parts for all doses delivered to the dosimeter vials within 24 days of the dosimeter storage. The measured thicknesses of the irradiated parts on day 24 were the same as those measured two hours after irradiation. The two photos in Figure 5a,b were superimposed (not shown here) to test if the irradiated parts are overlapped and if they were the same size and shape after 24 days of storage. The effect was that the irradiated parts recorded two hours and 24 days after irradiation matched very well. In summary, PABIG^nx^ maintains an integral 3D dose distribution for 24 days after irradiation.

Two dosimeter vials were irradiated to doses within the linear dose range of PABIG^nx^, however, one was irradiated above the dose linear range. This was done deliberately to investigate the effect of the increase in opacity at the edges of the irradiated parts of the dosimeter [1]. Some dosimeters showed this effect due to the slow diffusion of the bulk monomeric components to the edges of the irradiated regions, when the monomers in the irradiated region completely converted into cross-linked polymer, however, the dosimeter was still irradiated. In such case, water radiolysis still takes place, and the radicals may form on the polymer chains during ongoing irradiation. Diffusing bulk monomer molecules may react with the water radiolysis products or the radicals on the polymer chains. This increases the opacity at the edges of the irradiated parts, which has a negative effect on the dosimeter. Current study has not revealed this behaviour for monomer molecules at doses used in both a linear and over the linear range. Undoubtedly, however, further studies using magnetic resonance imaging [1] would enrich and probably support the observations in the present study. It should also be emphasized that for doses higher than the saturation dose, the cross-linked polymer chains in PABIG^nx^ may be susceptible to degradation. It is well-known that in the case of polymerisation induced by ionising radiation, simultaneous degradation of growing polymer chains may occur [19]. Supposedly, this would reduce the opacity of the irradiated regions. However, naked eye observations did not show a decrease in opacity for the dose over the saturation dose (Figure 5a,b); the pixel intensity was equal to 76.3, 122.5, 136.7 and 145.5 for 0, 10, 15 and 40 Gy (Figure 5a). Moreover, if the degradation took place at higher doses, the R_2_ = f(dose) relationship (Figure 4a) would deviate from the current one at doses higher than 30 Gy, so that R_2_ values would decrease with increasing dose. This has not been observed in the present study. In summary, it is likely that the 3D dose distribution recorded by PABIG^nx^ is stable for at least 24 days after irradiation, with no degradation observed for doses up to 40 Gy and possibly no enhancement of the dose-effect at the edges of the irradiated parts.

### 3.6. Tissue/Water Equivalence

**Figure 6:**
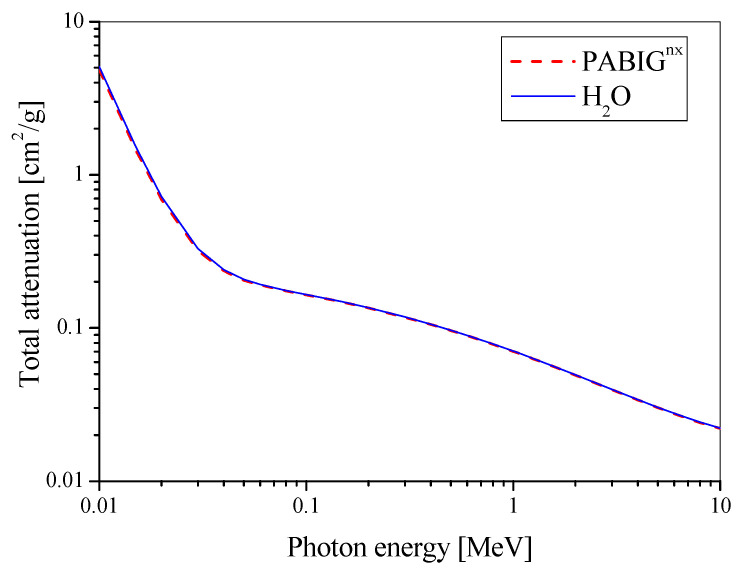
Total attenuation coefficient without coherent scattering as a function of the photon energy for water and PABIG^nx^.

The water equivalence, expressed as effective atomic number (Z_eff_), elemental composition, physical density (ρ), atomic-to-mass number ratio (<Z/A>) and electron density (ρ <Z/A>), of PABIG^nx^ was calculated as described in a previous work [24]. The data is presented in Table 2. Additionally, the total attenuation coefficient without coherent scattering as a function of the photon energy for water and PABIG^nx^ is shown in Figure 6. This is clear that PABIG^nx^ is water and soft tissue equivalent in terms of interaction with ionising radiation. This is in line with previous findings [24].

## 4. Conclusions

This work reports the basic characteristics of the PABIG^nx^ polymer gel dosimeter. This dosimeter responds linearly to a dose from 0.5 to ~18 Gy, saturates at about 40 Gy and has a dose sensitivity of 0.071 ± 0.001 Gy^−1^ s^−1^ (irradiation: clinical linear accelerator in a water phantom; measurement: 0.5 T NMR). It is also stable for at least eleven days after irradiation. The recorded 3D dose distribution is stable for 24 days after irradiation (maximum period of measurements in this study). PABIG^nx^ dose-response is independent of the irradiation with 6 MV photons, 15 MV photons, 6 MV photons FFF of 0.0168–0.1094 Gy/s dose rates. The dosimeter is soft tissue equivalent (water, mussels). The adopted procedure of manufacturing, irradiation and measurements results in reproducible calibration characteristics of the dosimeter.

The outcomes of this study enriched the knowledge on the use of PABIG^nx^ for 3D radiotherapy dosimetry in that the dosimeter can be applied, for example, in the verification of tumors eradication plans.

## Figures and Tables

**Table 1:** Basic calibration features of PABIG^nx^ polymer gel dosimeter. Linear regression was calculated: R_2_ = a × D + R_2,0_, where R_2,0_ is the intercept, D is the absorbed dose, and a is the slope, which stands for the dose sensitivity of the dosimeter.

Dose Sensitivity [Gy^−1^ s^−1^]	Intercept [s^−1^]	R^2^	Threshold Dose [Gy]	Linear Dose Range [Gy]	Dynamic Dose Range [Gy]
0.070 ± 0.001	1.14 ± 0.03	0.991	0.5 *	0.5 *–~18	0.5 *–~40

* The lowest dose applied in this study.

**Table 2:** Elemental composition and tissue equivalence of PABIG^nx^ in reference to water.

	Elemental Composition [% by Weight]						ρ [g/cm^3^]	<Z/A>	ρ × <Z/A>	Z_eff_
	_6_C	_1_H	_16_O	_7_N	_29_Cu	_16_S				
PABIG^nx^	7.40	10.84	79.99	1.78	9.98 × 10^−7^	5.03 × 10^−7^	1.017 ± 0.002	0.554	0.563	7.39
Water	-	11.19	88.81	-	-	-	1.000	0.555	0.555	7.51

## Data Availability

The data supporting reported results are not stored in any publicly archived datasets. The readers can contact the corresponding author for any further clarification of the results obtained.
